# Explaining cocktail party effect and McGurk effect with a spiking neural network improved by Motif-topology

**DOI:** 10.3389/fnins.2023.1132269

**Published:** 2023-03-20

**Authors:** Shuncheng Jia, Tielin Zhang, Ruichen Zuo, Bo Xu

**Affiliations:** ^1^Institute of Automation, Chinese Academy of Sciences, Beijing, China; ^2^School of Artificial Intelligence, University of Chinese Academy of Sciences, Beijing, China; ^3^School of Information and Electronics, Beijing Institute of Technology, Beijing, China; ^4^Center for Excellence in Brain Science and Intelligence Technology, Chinese Academy of Sciences, Shanghai, China

**Keywords:** spiking neural network, Motif topology, reward learning, cocktail-party effect, McGurk effect

## Abstract

Network architectures and learning principles have been critical in developing complex cognitive capabilities in artificial neural networks (ANNs). Spiking neural networks (SNNs) are a subset of ANNs that incorporate additional biological features such as dynamic spiking neurons, biologically specified architectures, and efficient and useful paradigms. Here we focus more on network architectures in SNNs, such as the meta operator called 3-node network motifs, which is borrowed from the biological network. We proposed a Motif-topology improved SNN (M-SNN), which is further verified efficient in explaining key cognitive phenomenon such as the cocktail party effect (a typical noise-robust speech-recognition task) and McGurk effect (a typical multi-sensory integration task). For M-SNN, the Motif topology is obtained by integrating the spatial and temporal motifs. These spatial and temporal motifs are first generated from the pre-training of spatial (e.g., MNIST) and temporal (e.g., TIDigits) datasets, respectively, and then applied to the previously introduced two cognitive effect tasks. The experimental results showed a lower computational cost and higher accuracy and a better explanation of some key phenomena of these two effects, such as new concept generation and anti-background noise. This mesoscale network motifs topology has much room for the future.

## 1. Introduction

Spiking neural networks (SNNs) are considered the third generation of artificial neural networks (ANNs) (Maass, 1997). The biologically plausible network architectures, learning principles, and neuronal or synaptic types of SNNs make them more complex and powerful than ANNs (Hassabis et al., 2017). It has been reported that even a single cortical neuron with dendritic branches needs at least a 5-to-8-layer deep neural network for finer simulations (Beniaguev et al., 2021), whereby non-differential spikes and multiply-disperse synapses make SNNs powerful on tools for spatially-temporal information processing. In the field of spatially-temporal information processing, there has been much research progress significant amounts of research into SNNs for auditory signal recognition (Shrestha and Orchard, 2018; Sun et al., 2022) and visual pattern recognition (Wu et al., 2021; Zhang M. et al., 2021).

This paper highlights two fundamental elements of SNNs and the main differences between SNNs and ANNs: specialized network design and learning principles. The SNNs encode spatial information using fire rate and temporal information using spike timing, providing hints and inspiration that SNNs can integrate into visual and audio sensory data.

For the network architecture, specific cognitive topologies developed *via* evolution are highly sparse and but efficient in SNNs (Luo, 2021), whereas equivalent ANNs are densely recurrent. Many researchers attempt have tried to understand the biological nature of efficient multi-sensory integration by focusing on the visual and auditory pathways in biological brains (Rideaux et al., 2021). These structures are adapted for some specific cognitive functions, e.g., efficient actions. For example, an impressive sparse network filtered from the *C. Elegans* connectome can outperform other dense networks during reinforcement learning of the Swimmer task. Some biological discoveries can further promote the research development of structure-based artificial operators, including but not limited to lateral neural interaction (Cheng et al., 2020), the lottery hypothesis (Frankle and Carbin, 2018), and meta structure of network motif (Hu et al., 2022; Jia et al., 2022). ANNs using these structure operators can then be applied in different spatial or temporal information processing tasks, such as image recognition (Frankle et al., 2019; Chen et al., 2020), auditory recognition, and heterogeneous graph recognition (Hu et al., 2022). Furthermore, when only focusing on the learning of weight, the weight agnostic neural network (Gaier and Ha, 2019; Aladago and Torresani, 2021) is a representative of the methods that only train the connections instead of weights.

For the learning principles, SNNs are more tuned affected by learning principles from biologically plausible plasticity principles, such as spike-timing dependent plasticity (STDP) (Zhang et al., 2018a), short-term plasticity (STP) (Zhang et al., 2018b), and reward-based plasticity (Abraham and Bear, 1996), instead of by the pure multi-step backpropagation (BP) (Rumelhart et al., 1986) of errors in ANNs. The neurons in SNNs will be activated once the membrane potentials reach their thresholds, which makes them energy efficient. SNNs have been successfully applied on to visual pattern recognition (Diehl and Cook, 2015; Zeng et al., 2017; Zhang et al., 2018a,b, 2021a,b), auditory signal recognition (Jia et al., 2021; Wang et al., 2023), probabilistic inference (Soltani and Wang, 2010), and reinforcement learning (Rueckert et al., 2016; Zhang D. et al., 2021).

**Figure 1:**
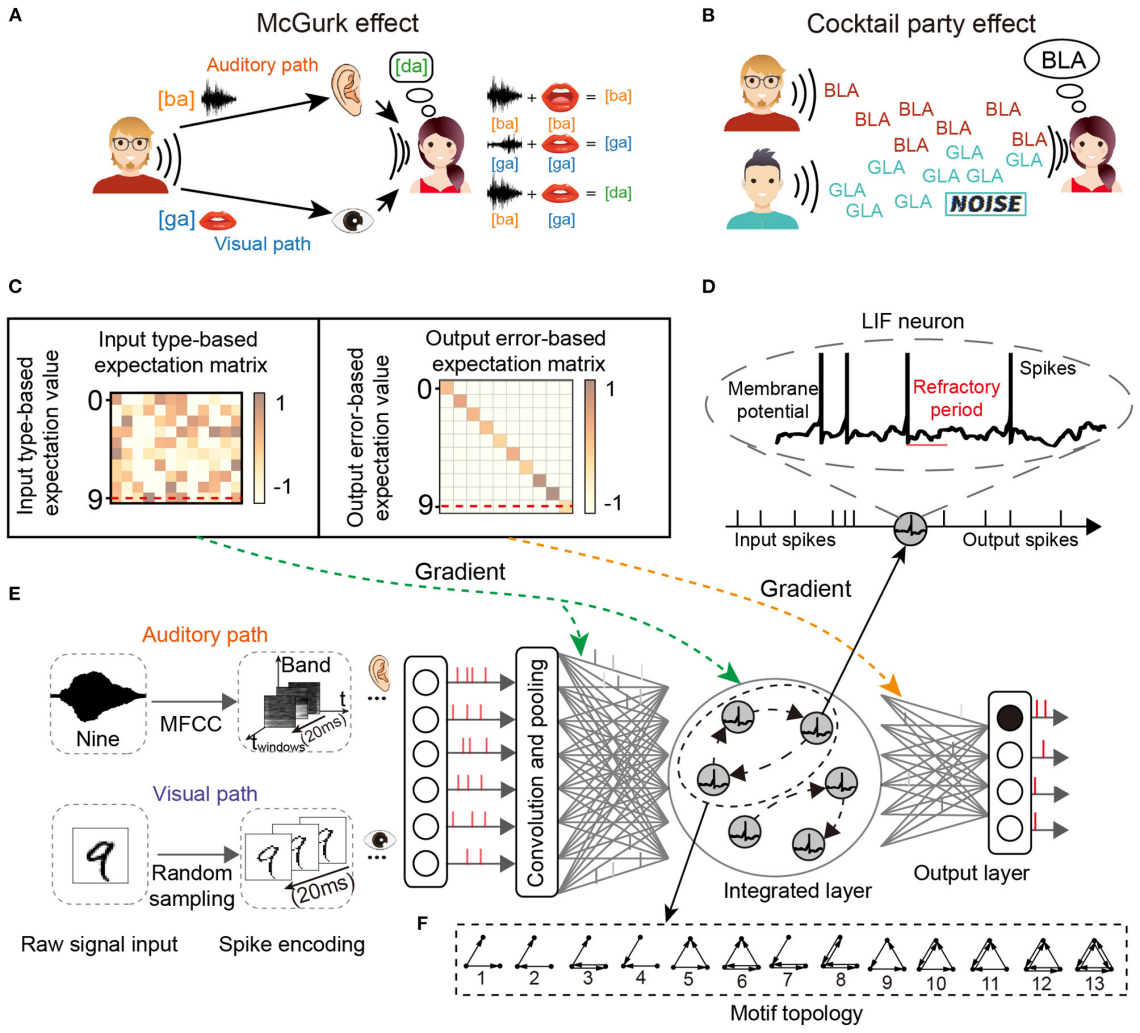
The network structure for multi-sensory integration and two cognitive phenomena. **(A)** McGurk effect. New concepts arise when the receiver receives different audio-visual input information. **(B)** Cocktail party effect. When the receiver's brain focuses on one speaker, it filters out the sounds and noise from others. **(C)** Input and output transformation matrix of reward learning. **(D)** The spiking neuron has a variable membrane potential. **(E)** The M-SNN network for single-sensory or multi-sensory integration tasks. **(F)** The example of 3-node Motifs.

For the two classic cognitive phenomena, the cocktail party effect describes the phenomenon that in a high-noise environment (e.g., noise from the environment or other speakers), the listener learns to filter out the background noise (including music noise and sounds from other speakers) and concentrate on only the target speaker, as shown in Figure 1A. The McGurk effect introduces the concept that the voice may be misclassified when the auditory stimulus conflicts with visual cues. A classic example of the McGurk effect describes how the new concept [da] can be generated by the integration of specific auditory input [ba] and visual cues [ga], as shown in Figure 1B.

This work focuses on the key characteristics of SNNs in information integration, categorization, and cognitive phenomenon simulation. We analyzed Motifs (Milo et al., 2002) in SNNs to reveal the essential functions of key meta-circuits in SNNs and biological networks and then used Motif structures to build loop modules in SNNs. Furthermore, a Motif-topology improved SNN (M-SNN) is proposed for simulating cocktail party effects and McGurk effects. To the best of our knowledge, we are the first to solve the problem using combinations of highly abstract Motif units. The following are the primary contributions of this paper:

- Networks with specific spatial or temporal types of Motifs can improve the accuracy of spatial or temporal classification tasks compared with networks without Motifs, making the multi-sensory integration easier by integrating two types of Motifs.
- We propose a method to mix different Motif structures and use them to simulate cognitive phenomena, including cocktail party effects and McGurk effects. In addition, the Motif topologies are critical, and networks with Motifs could effectively simulate these two effects (higher accuracy and better cognitive phenomenon simulation). (We specifically picked the MNIST and TIDigits datasets to simulate audio-visual inputs due to the lack of audio-visual-consistent datasets for classification testing.)
- During the network training process for various simulation experiments, the M-SNN can achieve a lower training computational cost than other SNNs without using Motif architectures. This result demonstrates that the M-SNN can achieve more human-like cognitive functions at a lower computational cost with the help of prior knowledge of multi-sensory pathways and biologically inspired reward learning methods.

The remaining parts are grouped as follows: Section 2 reviews the research about on the architecture, learning paradigms, and two classic cognitive phenomena. Section 3 describes the pattern of Motifs, the SNN model with neuronal plasticity, and learning principles. Section 4 verifies the convergence, the advantage of M-SNN in simulating cognitive phenomena, and the computational cost. Finally, a short conclusion is given in Section 5.

## 2. Related works

For the architecture, the lateral interaction of neural networks, the lottery hypothesis, and the network motif circuits are novel operators in structure research. In the research on lateral interaction, most studies have taken the synapse as the basic unit, including the lateral interaction in the convolutional neural network (Cheng et al., 2020) or that in the fully connected network (Jia et al., 2021). However, these methods take synaptic connections as the basic unit and only consider learning effective structures without considering meta-structure composition.

Network motifs (Milo et al., 2002; Prill et al., 2005) use primary *n*-node circuit operators to represent the complex network structures. The feature of the network (e.g., visual or auditory pathways) could be reflected by the number of different Motif topologies, which is called Motif distribution. To calculate the Motif distribution, the first Motif tool is mfinder, which implements the algorithm of full enumeration (randomly picking the edges from the graph and counting the probability of *n*-node subgraphs). Then the FANMOD (Wernicke and Rasche, 2006) was introduced as a more efficient tool for finding reliable network motifs.

For learning paradigms, there are many methods have been proposed, such as the ANN-to-SNN conversion (i.e., directly training ANNs and then equivalently converting to SNNs; Diehl et al., 2015), proxy gradient learning (i.e., replacing the non-differential membrane potential at firing threshold by an infinite gradient value; Lee et al., 2016), and the biological-mechanism inspired algorithms [e.g., the SBP (Zhang et al., 2021a) which was inspired by the synaptic plasticity rules in the hippocampus, the BRP (Zhang et al., 2021b), which was inspired by the reward learning mechanism, and the GRAPES, that inspired by the synaptic scaling (Dellaferrera et al., 2022)]. Compared to other learning algorithms, biologically inspired algorithms are more similar to the process of how the human brain learns.

For the cocktail party effect, many effective end-to-end neural network models have been proposed (Ephrat et al., 2018; Chao et al., 2019; Hao et al., 2021; Wang et al., 2021). However, the analysis of why these networks work is very difficult since the functional structures in these black-box models are very dense without clear function diversity. As a comparison, the network motif constraint in neural networks might resolve this problem to some extent, which until now and as far as we know, however this has not yet been well-introduced.

For the McGurk effect, only a limited number of research papers have discussed the artificial simulation of it, partly caused by the simulation challenge, especially on the conflict fusion of visual and auditory inputs (McGurk and MacDonald, 1976; Hirst et al., 2018), e.g., self-organized mapping (Gustafsson et al., 2014).

## 3. Methods

### 3.1. Spiking dynamics

The leaky integrated-and-fire (LIF) neuron model is biologically plausible and is one of the simplest models to simulate spiking dynamics. It includes non-differential membrane potential and the refractory period, as shown in Figure 1D. The LIF neuron model simulates the neuronal dynamics with the following steps.

First, the dendritic synapses of the postsynaptic LIF neuron will receive presynaptic spikes and convert them to a postsynaptic current (*I*_*syn*_). Second, the postsynaptic membrane potential will be leaky or integrated, depending on its historical experience. The classic LIF neuron model is shown as the following Equation (1).


(1) $$\begin{array}{l} \tau_{m}\frac{dV_{t}}{dt}=-\left(V_{t}-V_{L}\right)-\frac{g_{E}}{g_{L}}\left(V_{t}-V_{E}\right)+\frac{I_{syn}}{g_{L}}, \end{array}$$

where *V*_*t*_ represents the dynamical variable of membrane potential with time *t*, *dt* is the minimal simulation time slot (set as 0.01ms), τ_*m*_ is the integrative period, *g*_*L*_ is the leaky conductance, *g*_*E*_ is the excitatory conductance, *V*_*L*_ is the leaky potential, *V*_*E*_ is the reversal potential for excitatory neuron, and *I*_*syn*_ is the input current received from the synapses in the previous layer. We set values of conductance (*g*_*E*_, *g*_*L*_) to be 1 in our following experiments for simplicity, as shown in Equation (3).

Third, the postsynaptic neuron will generate a spike once its membrane potential *V*_*t*_ reaches the firing threshold *V*_*th*_. At the same time, the membrane potential *V* will be reset as the reset potential *V*_*reset*_, shown as the following Equation (2).


(2) $$\text{ if }\left(V_{t}>V_{th}\right)\{\begin{array}{l} V_{t}=V_{reset}\\ T_{ref}=T_{0} \end{array},$$

where the refractory time *T*_*ref*_ will be extended to a larger predefined *T*_0_ after firing.

In our experiments, the three steps for simulating the LIF neurons were integrated into the Equation (3).


(3) $$\begin{array}{l} C\frac{dV_{i,t}}{dt}=g\left(V_{i,t}-V_{rest}\right)\left(1-S_{i,t}\right)+\overset{N}{\underset{j\;=\;1}{\sum }}W_{i,j}X_{j,t}, \end{array}$$

where *C* is the capacitance parameter, *S*_*i,t*_ is the firing flag of neuron *i* at timing *t*, *V*_*i,t*_ is the membrane potential of neuron *i* at timing *t*, *V*_*rest*_ is the resting potential, and *W*_*i,j*_ represents the synaptic weight between the neuron *i* and *j*.

### 3.2. Motif topology

**Figure 2:**
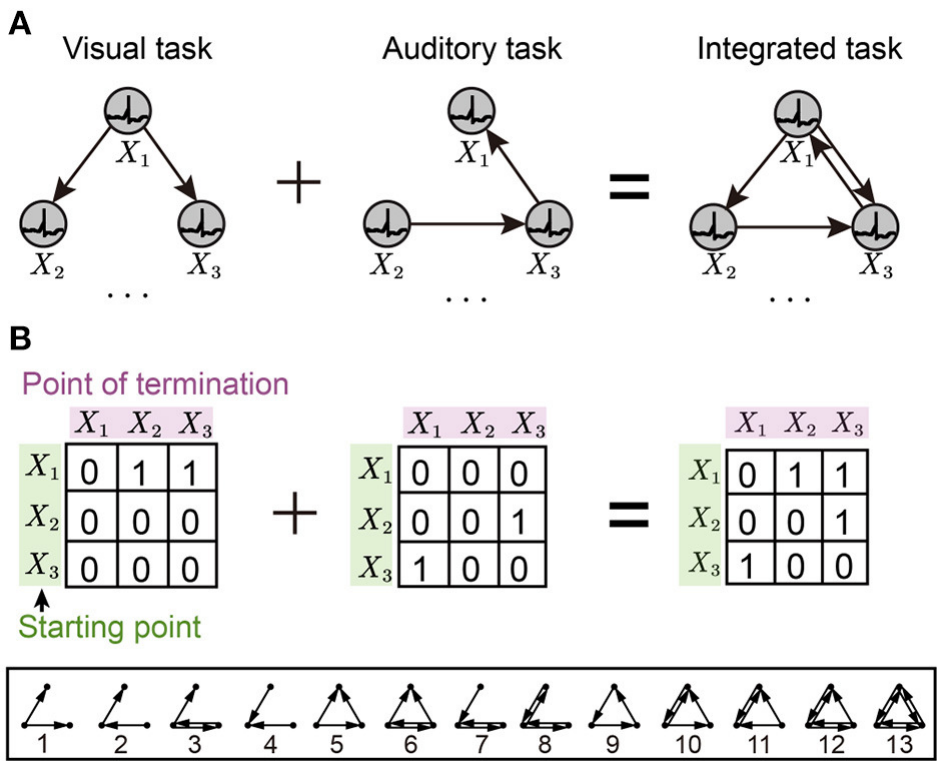
Schematic diagram of an example for integrating Motif masks. **(A)** Schematic for Motifs of the M-SNN. **(B)** Schematic for Motif masks of the M-SNN.

The *n*-node (*n* ≥ 2) meta Motifs have been proposed in past research. Here, we use the typical 3-node Motifs to analyze the networks, which have been widely used in biological and other systems (Milo et al., 2002; Shen et al., 2012; Zhang et al., 2017). Figure 1F displayed all 13 varieties of 3-node Motifs. In previous studies, network topology had been transformed into parameter embeddings in the network (Liu et al., 2018). In our SNNs, the Motifs were used by the Motif masks and then applied into the recurrent connection at the hidden layer. The typical Motif mask is a matrix padded with 1 or 0, where 1 and 0 represent the connected and non-connected pathways, respectively. We introduce the Motif circuits into the hidden layer, and the Motif mask in the *r*-dimension hidden layer *l* at time *t* is represented as the $M_{t}^{r,l}$ as shown in Equation (4). As shown in Figure 2, we show some examples of Motifs (Figure 2A) and their corresponding Motif masks (Figure 2B). The Motif masks are generated by binary square matrices where only one (with connection) and zero (without connections) values are designed.


(4) $$M_{t}^{r,l}=\left[\begin{array}{ccc} f(m_{1,2}) & \cdots & f(m_{1,r})\\ \vdots & \ddots & \vdots \\ f(m_{r,1}) & \cdots & f(m_{r,r}) \end{array}\right],$$

where *f*(·) is the indicator function. Once the variable in *f*(·) satisfies the conditions, the function value would be set as one; otherwise, zero. *m*_*i,j*_, (*i, j* = 1, ⋯*r*) are elements of synaptic weight $W_{t}^{r,l}$.

The network motif distribution is calculated by counting the occurrence frequency of network motif types. We enumerate every 3-node assembly (including Motifs and other non-Motif types) and only count the 13-type 3-node connected subgraphs of Motifs with the help of FANMOD (Wernicke and Rasche, 2006). In order to integrate the Motifs learned from different visual and auditory datasets, we propose a multi-sensory integration algorithm by integrating Motif masks with different types learned from visual or auditory classification tasks. Hence, the integrated Motif connections have both visual and auditory network patterns, as shown in Figure 2. Equation (5) shows the integrated equation with visual and auditory Motif masks.


(5) $$\begin{array}{l} M_{t}^{r,l}=M_{t}^{r,l}\left(s\right)\cup M_{t}^{r,l}\left(t\right), \end{array}$$

where $M_{t}^{r,l}\left(s\right)$ is the spatial mask that learned from the visual dataset, $M_{t}^{r,l}\left(t\right)$ is the temporal mask that learned from the auditory dataset, and $M_{t}^{r,l}$ is the integrated mask. “∪” means the OR operation for every element of the visual Motif mask and auditory Motif mask.

For forming the network motifs in SNN, the Motif mask is used to mask the lateral connections in the neural network. The lateral and sparse connections between LIF neurons are usually designed to generate network-scale dynamics. As shown in Figure 1E, we design a four-layer SNN architecture, containing an input layer (for pre-encoding visual and auditory signals to spike trains), a convolutional layer, a multi-sensory integration layer, and a readout layer. The synaptic weights are adaptive while the Motif masks are not. The membrane potentials in the hidden multi-sensory-integration layer are updated by both feed-forward potential and recurrent potential, shown in the following Equation (6):


(6) $$\left\{ \begin{array}{l} \begin{array}{c} S_{i,t}=S_{i,t}^{f}+S_{i,t}^{r} \end{array}\\ \begin{array}{c} V_{i,t}=V_{i,t}^{f}+V_{i,t}^{r} \end{array}\\ \begin{array}{cc} C\frac{dV_{i,t}^{f}}{dt}= & g(V_{i,t}-V_{rest})(1-S_{i,t})+\sum_{j=1}^{N}W_{i,j}^{f}X_{j,t} \end{array}\\ \begin{array}{c} C\frac{dV_{i,t}^{r}}{dt}=\sum_{j=1}^{N}W_{i,j}^{r}S_{i,t}\cdot M_{t}^{r,l} \end{array} \end{array}\right.,$$

where *C* is for capacitance, *S*_*i,t*_ is the firing flag of neuron *i* at time *t*, $S_{i,t}^{f}$ and $S_{i,t}^{r}$ are the firing flags of neuron *i* in the feedforward process and recurrent process, respectively, *V*_*i,t*_ denotes the membrane potential of neuron *i* at timing *t*, which includes feed-forward $V_{i,t}^{f}$ and recurrent $V_{i,t}^{r}$, *V*_*rest*_ is the resting potential, $W_{i,j}^{f}$ is the feed-forward synaptic weight from the neuron *i* to the neuron *j*, and $W_{i,j}^{r}$ is the recurrent synaptic weight from the neuron *i* to the neuron *j*. $M_{t}^{r,l}$ is the mask incorporating Motif topology to further alter feed-forward propagation further. The historical information is saved in the forms of recurrent membrane potential $V_{i,t}^{r}$, where spikes are created after the potential reaches a firing threshold, as illustrated in Equation (7).


(7) $$\{\begin{array}{l} V_{i,t}^{f}=V_{reset},S_{i,t}^{f}=1\;if(V_{i,t}^{f}=V_{th})\\ V_{i,t}^{r}=V_{reset},S_{i,t}^{r}=1\;if\left(V_{i,t}^{r}=V_{th}\right)\\ S_{i,t}^{f}=1\;if\left(t-t_{s^{f}}<\tau_{ref},t\in \left(1,T_{1}\right)\right)\\ S_{i,t}^{r}=1\;if\left(t-t_{s^{r}}<\tau_{ref},t\in \left(1,T_{2}\right)\right) \end{array},$$

where $V_{i,t}^{f}$, $V_{i,t}^{r}$, $S_{i,t}^{f}$, and $S_{i,t}^{r}$ are introduced in the previous Equation (6). *V*_*reset*_ is the reset membrane potential. τ_*ref*_ is the refractory period. $t_{s^{f}}$ is the previous feed-forward spike timing and $t_{s^{r}}$ is the previous recurrent spike timing. *T*_1_ and *T*_2_ are time windows.

### 3.3. Neuronal plasticity and learning principle

We use three key mechanisms during network learning: neuronal plasticity, local plasticity, and global plasticity.

Neuronal plasticity emphasizes spatially-temporal information processing by considering the inner neuron dynamic characteristics (Jia et al., 2021), different from traditional synaptic plasticities such as STP and STDP. The neuronal plasticity for SNNs approaches the biological network and improves the learning power of the network. Rather than being a constant value, the firing threshold is set by an ordinary differential equation shown as follows:


(8) $$\begin{array}{l} \frac{da_{i,t}}{dt}=\left(\alpha -1\right)a_{i,t}+\beta \left(S_{t}^{f}+S_{t}^{r}\right), \end{array}$$

where $S_{t}^{f}$ is the input spikes from the feed-forward channel. $S_{t}^{r}$ is the input spikes from the recurrent channel. *a*_*i,t*_ is the dynamic threshold, which has an equilibrium point of zero without input spikes or $-\frac{\beta }{\alpha -1}$ with input spikes *S*^*f*^ + *S*^*r*^ from the feed-forward and recurrent channels. Therefore, the membrane potential of adaptive LIF neurons is updated as follows:


(9) $$\begin{array}{l} \begin{array}{c} C\frac{dV_{i,t}}{dt}=g\left(V_{i,t}-V_{rest}\right)\left(1-S_{t}^{f}-S_{t}^{r}\right)+\overset{N}{\underset{j\;=\;1}{\sum }}W_{i,j}X_{j,t}-\gamma a_{i,t} \end{array}, \end{array}$$

where the dynamic threshold *a*_*i,t*_ is accumulated during the period from the resetting to the membrane potential firing and finally attains a relatively stable value $a_{i,t}^{*}=\frac{\beta }{1-\alpha }\left(S_{t}^{f}+S_{t}^{r}\right)$. Because of the −γ*a*_*i,t*_, the maximum firing threshold could reach up to *V*_*th*_ + γ*a*_*i,t*_.

We set α = 0.9 to guarantee that the coefficient of *a*_*i,t*_ is −0.1, β = 0.1 to ensure that the spike has the same weight as *a*_*i,t*_, and set γ to the common value of 1. Accordingly, the stable $a_{t}^{*}=0$ for no input spikes, $a_{t}^{*}=1$ for one input spike, and $a_{t}^{*}=2$ for input spikes from two channels. When $a_{i,t}<\left(S_{t}^{f}+S_{t}^{r}\right)$, the threshold *a*_*i,t*_ will increase, otherwise, the threshold *a*_*i,t*_ will decrease. It is clear that the threshold will change in the process of the neuron's firing, and as the firing frequency of the neuron increases, the threshold will also elevate, or vice versa.

For local plasticity, the membrane potential at the firing time is a non-differential spike, so local gradient approximation (pseudo-BP) (Zhang et al., 2021b) is usually used to make the membrane potential differentiable by replacing the non-differential part with a predefined number, shown as follows:


(10) $$Grad_{local}=\frac{\partial S_{i,t}}{\partial V_{i,t}}=\{\begin{array}{cc} 1 & if\left(\left|V_{i,t}-V_{th}\right|<V_{win}\right)\\ 0 & else \end{array},$$

where *Grad*_*local*_ is the local gradient of membrane potential at the hidden layer, *S*_*i,t*_ is the spike flag of neuron *i* at time *t*, *V*_*i,t*_ is the membrane potential of neuron *i* at time *t*, and *V*_*th*_ is the firing threshold. *V*_*win*_ is the range of parameters for generating the pseudo-gradient. This approximation makes the membrane potential *V*_*i,t*_ differentiable at the spiking time between an upper bound of *V*_*th*_ + *V*_*win*_ and a lower bound of *V*_*th*_ − *V*_*win*_.

For global plasticity, we used reward propagation, which has been proposed in our previous work (Zhang et al., 2021b). As shown in Figure 1C, the gradient of the hidden layer in training is generated from the input type-based expectation value and output error-based expectation value by transformed matrix (input type-based expectation matrix and output error-based expectation matrix), respectively, then the gradient signal will be directly given to all hidden neurons without layer-to-layer backpropagation, shown as follows:


(11) $$\{\begin{array}{l} Grad_{R_{l}}=B_{rand}^{f,l}\cdot R_{t}-h^{f,l}\\ Grad_{R_{L}}=B^{f,L}\cdot e^{f,L}\\ \Delta W_{t}^{f,l}=-\eta^{f}(Grad_{R_{l}})\\ \Delta W_{t}^{r,l}=-\eta^{r}\left(Grad_{t+1}+Grad_{R_{l}}\right)\cdot M_{t}^{r,l}\\ \Delta W_{t}^{f,L}=-\eta^{f}(Grad_{R_{L}}) \end{array},$$

where *h*^*f, l*^ is the current state of layer *l* and, *R*_*t*_ is the predefined input-type based expectation value. A predefined random matrix $B_{rand}^{f,l}$ is designed to generate the reward gradient *Grad*_*R*_*l*__. *Grad*_*R*_*L*__ is the gradient of the last layer, *B*^*f, L*^ is the predefined identity matrix, and *e*^*f, L*^ is the output error. $W_{t}^{f,l}$ represents the synaptic weight at layer *l* in feed-forward phase, $\Delta W_{t}^{r,l}$ is the recurrent-type synaptic modification at layer *l* which represents defined by both *Grad*_*R*_*l*__ by reward learning and *Grad*_*t*+1_ by iterative membrane-potential learning, and the *Grad*_*t*+1_ means the gradient obtained at *t* + 1 moment (Werbos, 1990). The $M_{t}^{r,l}$ is the mask incorporating Motif topology to influence the propagated gradients further.

### 3.4. The learning procedure of M-SNN

The overall learning procedures of the M-SNN were shown in Algorithm 1, including the raw signal encoding, Motif structure integration, and cognitive effect simulation.

**Algorithm 1:** The M-SNN algorithm.

1. Initialize the network by resetting weights and all related parameters. e.g., initial membrane potential *V*_*i*_, simulation time *T*, learning rates η = η^*f*^ = η^*r*^. 2. Encode raw numbers of datasets to spike trains. 3. Learn the synaptic weights *w*_*ij*_ and Motif masks $M_{t}^{r,l}$ by BP (Rumelhart et al., 1986) in two single-sensory tasks to get the spatial mask $M_{t}^{r,l}\left(s\right)$ and temporal mask $M_{t}^{r,l}\left(t\right)$. 4. Synthesize Motif masks and train the synaptic weight *w*_*ij*_ on multi-sensory integration tasks. 4.1 Synthesize the integrated masks $M_{t}^{r,l}$ from spatial and temporal masks, where $M_{t}^{r,l}=M_{t}^{r,l}\left(s\right)\cup M_{t}^{r,l}\left(t\right)$. 2%. 4.2 Initialize a new network and embed the Motif mask $M_{t}^{r,l}$. 2%. 4.3 Only learn the synaptic weight *w*_*ij*_ with local Pseudo-BP and global reward learning (Zhang et al., 2021b). 5. Test the performance of SNNs using these new masks in the multi-sensory classification tasks and simulate the cocktail party effect and McGurk effect.

## 4. Experiments

### 4.1. Visual and auditory datasets

The MNIST dataset (LeCun, 1998) was selected as the visual sensory dataset. The MNIST dataset contains 60,000 28 × 28 one-channel grayscale images of handwritten digits from zero to nine for training, and there are also 10,000 of the same type of data for testing. The TIDigits dataset (Leonard and Doddington, 1993) was selected as the auditory sensory dataset, containing 4,144 spoken digit recordings from zero to nine. Each recording was sampled at 20 kHz for around one second and then transformed to the frequency domain with 28 frames and 28 bands by the Mel Frequency Cepstral Coefficient (MFCC) (Sahidullah and Saha, 2012). Some examples were shown in Figure 1E.

### 4.2. Experimental configurations

The SNNs were built in Pytorch, and the network architectures for MNIST and TIDigits were the same, containing one input encoding layer, one convolutional layer (with a kernel size of 5 × 5, and two input channels constructed by convolutional layer), one full-connection integrated layer (with 200 LIF neurons), and one output layer (with ten output neurons). Among the network, the capacitance *C* was 1μF/cm^2^, conductivity *g* was 0.2 nS, time constant τ_*ref*_ was 1 ms, and resting potential *V*_*rest*_ was equal to reset potential *V*_*reset*_ with 0 mV. The learning rate was 1*e*-4, the firing threshold *V*_*th*_ was 0.5 mV, the simulation time *T* was set as 20 ms, and the gradient approximation range *V*_*win*_ was 0.5 mV.

As shown in Figure 1E, for the visual dataset, before being given to the input layer, the raw data were encoded to spike trains first by comparing each number with a random number generated from Bernoulli sampling at each time slot of the time window *T*. For the auditory dataset, the input data would first be transformed to the frequency spectrum in the frequency domain by the MFCC (Mel frequency cepstrum coefficient; Sahidullah and Saha, 2012). Then the spectrum would be split according to the time windows. Finally, the sub-spectrum would be converted into normalized value and randomly sampled with Bernoulli sampling to spike trains.

There are two SNNs concluded in our experiment as follows:

- M-SNN. The Motif mask is generated randomly and then updated during the learning of synaptic weights in a Standard-SNN.
- Standard-SNN. The standard feed-forward SNN without Motif masks acts as the control algorithm for comparing M-SNN.

### 4.3. Analysis of spatial and temporal Motif topology during learning

**Figure 3:**
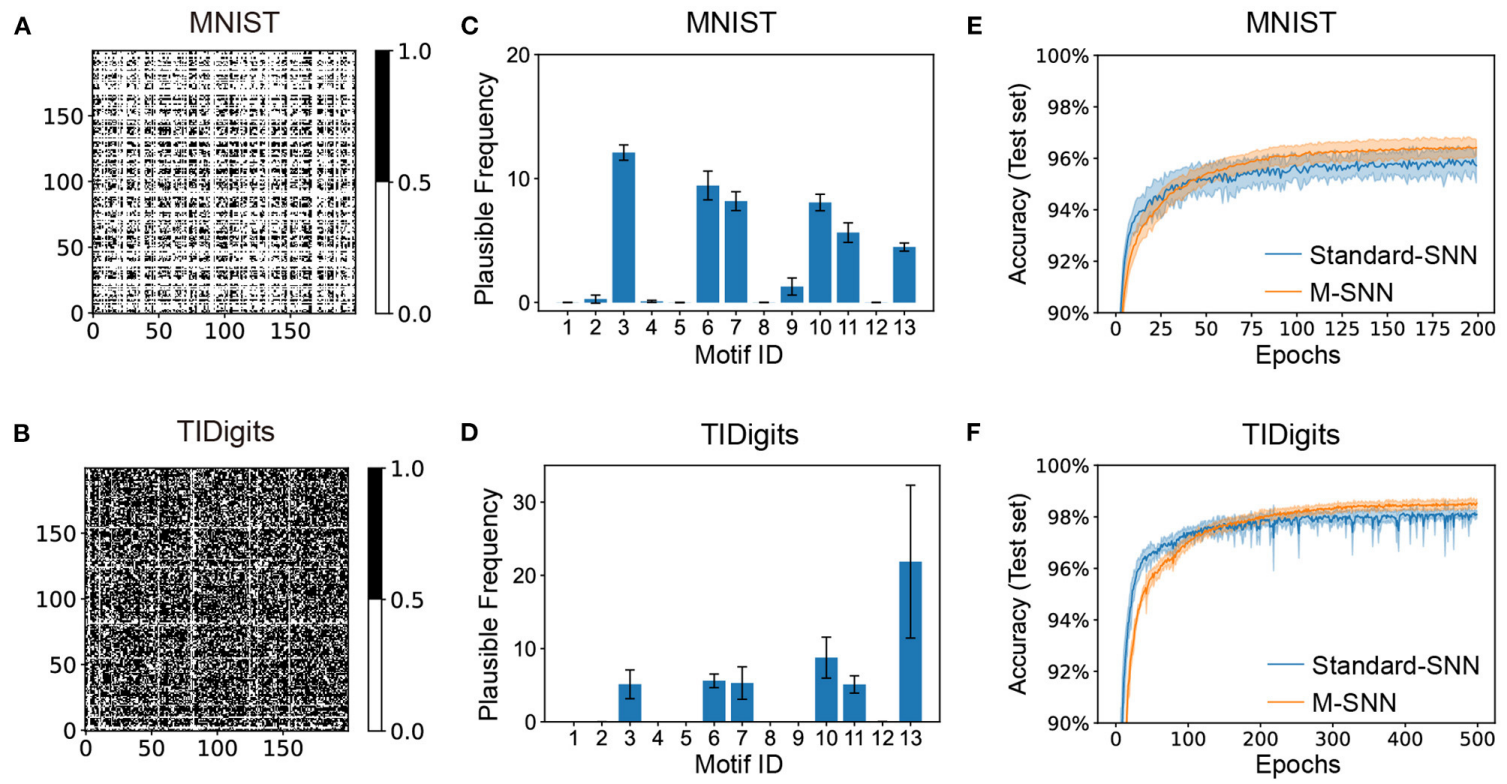
Network convergence with SNNs using Motifs in different datasets. **(A, B)** Motif masks of MNIST **(A)** and TIDigits **(B)** after training. **(C, D)** Plausible Frequency of Motif distributions of MNIST **(C)** and TIDigits **(D)** datasets after training. **(E, F)** Convergence curve of classification task of MNIST **(E)** and TIDigits **(F)** datasets.

The visual and auditory Motif masks were shown in Figure 3, which were trained from the MNIST and TIDigits datasets. After training, the generated visual and temporal Motif masks were shown in Figures 3A, B, where the black dot in the visualization of the Motif mask indicated that there was a connection between the two neurons shown at the X-axis and Y-axis. The white dot meant there was not.

This result showed that the visual Motif mask connections were sparse, with only about half of the neurons being connected. Furthermore, the connection in the Motif mask is 64.39% for auditory TIDigits dataset, and 28.24% for visual MNIST dataset. For the temporal TIDigits dataset, the generated temporal Motif mask after training was shown in Figure 3B, where the learned Motif mask was denser than that on the visual MNIST in Figure 3A. It is consistent with the biological finding that temporal Motifs are denser than visual ones (Vinje and Gallant, 2000; Hromádka et al., 2008). These differences between spatial and temporal Motif masks indicated that the network needed a more complex connection structure to deal with sequential information. In addition, the connection points in the spatial and temporal Motif masks in Figures 3A, B seemed to be divided into several square regions, similar to the brain regions, which, to some extent, shows the similarity between artificial and biological neural networks at the brain region scale.

The information presented by Motif masks is relatively limited. For further analysis of the Motif structures by Motif distribution, we used the “Plausible Frequency” instead of the standard frequency to calculate the significant Motifs after comparing them to the random networks. The “Plausible Frequency” was defined by multiplying the occurrence frequency and 1−*P*, where the *P* was the P-value of a selected Motif after comparing it to 2,000 repeating control tasks with random connections. The “repeating control tasks” meant generating many matrixes (e.g., 2000) that each element was sampled from a uniformly random distribution. Furthermore, the *P*-value index showed the statistical significance of the concerning results, whereas a lower *P*-value indicated the more plausible result.

The Motif distributions corresponding to the Motif masks were shown in Figures 3C, D, where the spatial and temporal Motifs were distributed differently. For spatial Motifs, the 3rd, 6th, 7th, and 10th units were all prominent in spatial Motifs, while the 13th Motif was the most prominent in temporal Motifs. The abundant 3rd, 6th, 7th, and 10th Motifs in SNN revealed the balance of feedforward and recurrent connections for the spatial tasks. The Motif distribution reveals the difference in the abundance of micro-loops in different networks, indicating that temporal tasks require more complex network connections than spatial tasks. To some extent, the Motif distribution here can mitigate the “black box” problem of ANNs by clearly showing loop-level network differences. The plausible frequency eliminated the interference from the random connection. Figures 3E, F showed that M-SNN networks using Motif topologies can be convergent, where the accuracy of M-SNN was significantly higher than the accuracy of Standard-SNN after a few training epochs.

### 4.4. M-SNN contribute to solving the cocktail party effect

The cocktail party effect consists of two conditions. The first condition involves focusing on one person's conversation and excluding other conversations or noise in the background. Second, it refers to the response of our hearing organs to a certain stimulus. The human attention mechanism has much to do with how the cocktail party effect happens. In our SNN, we simulated the first situation of the cocktail party effect. We used the MNIST dataset to represent the visual input and the TIDigits dataset for the phonetic input. We modeled two scenes to simulate the simplified cocktail party effect. The first scene was a simulation of the cocktail party effect, where both the visual and auditory inputs were messed up by random noise. The second scene simulated a cocktail party effect in which the visual and auditory inputs were simultaneously disrupted by the real image and voice.

#### 4.4.1. Visual and auditory inputs are interfered with the stochastic noise

**Figure 4:**
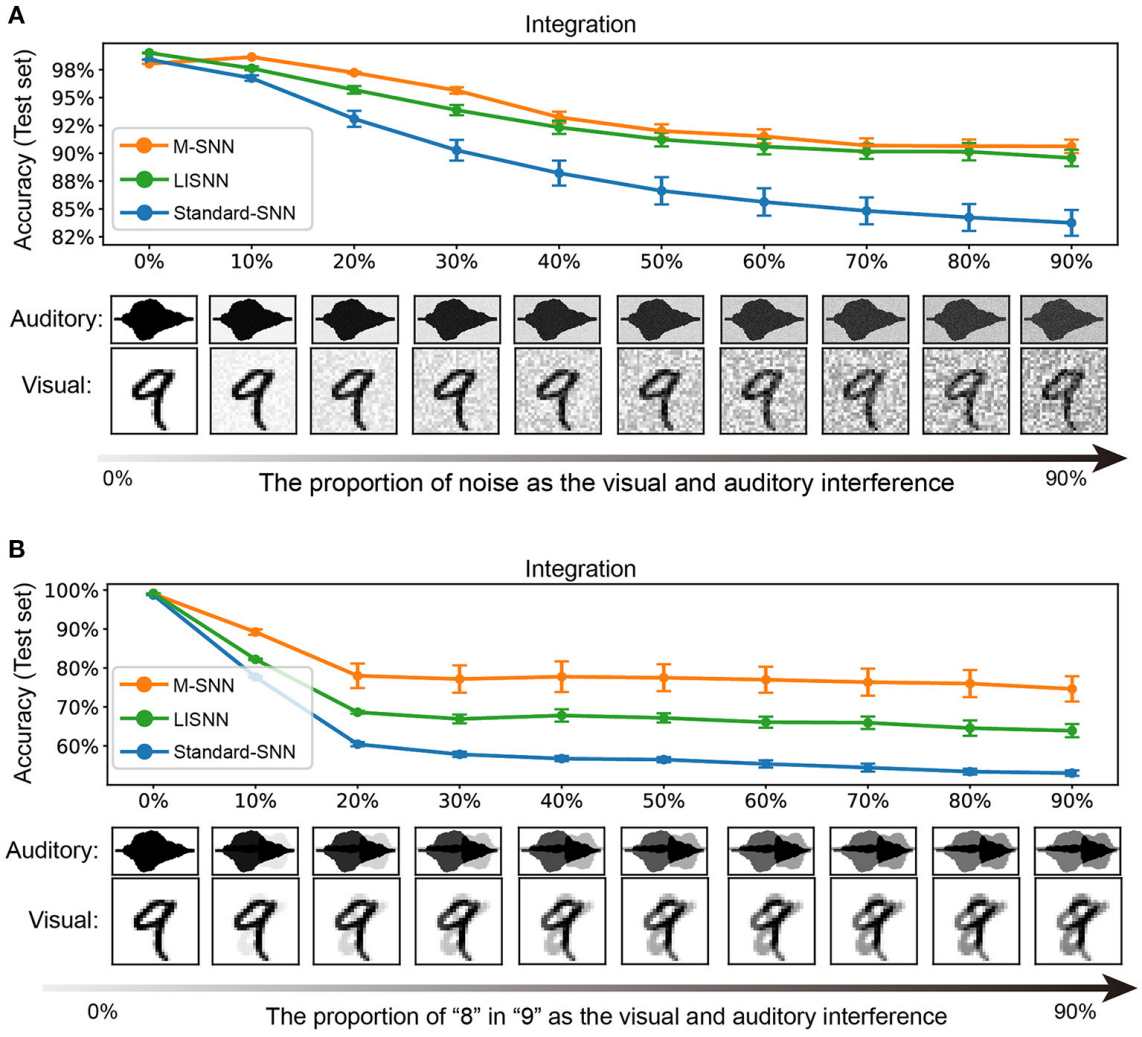
Simulation of cocktail party effect. **(A)** A simulation and results in which both visual and auditory inputs have interfered. **(B)** A simulation and results in which only the voice has interfered. All figures are averaged over five repeating experiments with different random seeds.

In our experiment, we trained the network with pure image and voice inputs and tested the network with input disturbed by stochastic noise. In the simulation process, we used the method of superimposing random numbers between [0, 1] into the image or speech input to simulate the interference effect of noise. With the different values of the added random numbers, different interference effects were formed, ranging from 0 to 90%, and the influence gradually increased. As shown in Figure 4A, when the influence of noise was relatively low, whether to adding Motifs into the network had little effect on the experimental results (99.00 ± 0.00% for the network with Motifs, 98.50 ± 0.22% for Standard-SNN, and 99.14 ± 0.03% for LISNN; Cheng et al., 2020). As shown in Figure 4A, with the increase of noise ratio, the recognition ability of the network to the input target signal decreased gradually. When the proportion of noise was increased to 60%, the accuracy of the M-SNN was 95.64 ± 0.29%, which was markedly higher than the accuracy of Standard-SNN (57.84 ± 0.68%) and was comparable with LISNN (93.88 ± 0.46%). The higher accuracy indicated that the Motifs in M-SNN had a positive effect on solving the cocktail party effect compared with Standard-SNN. Furthermore, LISNN with lateral interaction in the convolution layer could get a comparable effect with M-SNN.

#### 4.4.2. Visual and auditory inputs are interfered with the real image and voice

We used the MNIST and TIDigits datasets without noise when training the network. We used “8” from the handwritten digital image and human voice in the simulation process instead of the stochastic noise as interference. As shown in Figure 4B, in the case of a few other interfering sounds, the effect of M-SNN on maintaining accuracy was insignificant. However, with the increase in the proportion of different interfering sounds, the impact of M-SNN on maintaining the recognition of the network was becoming more and more significant. When the noise ratio reached 50%, the recognition accuracy of M-SNN became 77.77 ± 3.94 %, while the Standard-SNN could only reach the an accuracy of 56.75 ± 0.67%, and the accuracy achieved by LISNN was 67.83 ± 1.58%. In these situations, the maximal increased accuracy was 7.5% when the proportion of “8” was 50%.

### 4.5. M-SNN for explainable McGurk effect

The McGurk effect described the psychological phenomenon that occurs when human speech input and image input are inconsistent, whereby most people would judge the input as neither a speech label nor a visual label but a novel concept. It had been shown that, for adults, the error rate in judging inconsistent audio-visual input as novel concepts was more than 90% (McGurk and MacDonald, 1976). For example, when the speech input was [ba] and the visual input was [ga], a new concept [da] was generated (Tiippana, 2014). During the simulation, we used handwritten digit images [2],[3] as the visual input, while speech digits [*tu*:],[θ*ri*:] were used to represent the corresponding pronunciation.

**Figure 5:**
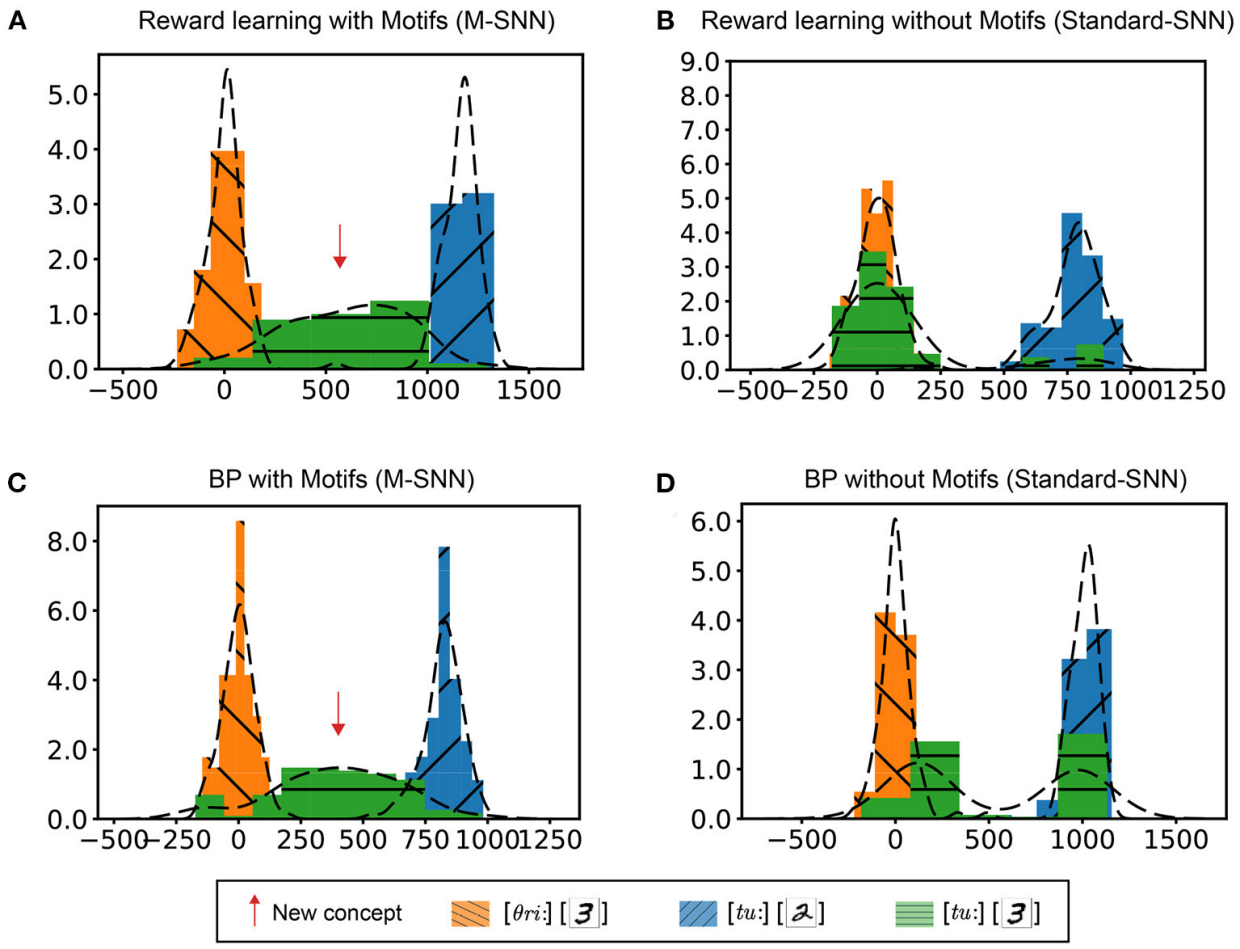
Simulation of McGurk effect. **(A, B)** Distribution in the integrated layer after reward learning with Motifs **(A)** and without Motifs **(B)** of different combinations of input. **(C, D)** Distribution in integrated layer after BP learning with Motifs **(C)** and without Motifs **(D)** of different combinations of input.

First, consistent audio-visual inputs were used to train the network weights. After training, the inconsistent audio-visual information would be fed into the network. In the integrated layer, we used TSNE (Maaten and Hinton, 2008) to reduce the dimension of the high-dimensional features. We conducted four experiments to verify the influence of learning rules and structures on the McGurk effect simulation: networks trained with reward learning with Motif (Figure 5A), networks trained with reward learning without Motif (Figure 5B), networks trained with BP learning with Motif (Figure 5C), and networks trained with BP without Motif (Figure 5D). As shown in Figure 5, the histogram showed the distribution of samples with different labels in the integration layer. The *x*-axis represents the distance between the feature point and the reference point on the 2D plane (using TSNE for clustering). For the Standard-SNN, there were two prominent feature distributions: [θ*ri*:,3] and [*tu*:,2]. However, for the learning results of M-SNNs, a clear feature distribution of [*tu*:,3] emerged between the distributions of [θ*ri*:,3] and [*tu*:,2]). This distribution corresponding to [*tu*:,3] characterized the new concept (McGurk effect). These results showed that Motifs in SNNs are important for generating the McGurk effect, and neither of these learning principles alone can produce the McGurk effect.

For comparing the stimulating effect of the McGurk effect, we compared additional algorithms as shown in Table 1. According to our knowledge, the SOM approach in the paper (Gustafsson et al., 2014) is the only unsupervised learning method that replicates the McGurk effect. In contrast, our M-SNN is the only supervised learning method.

**Table 1:** Performance of different algorithms on simulating McGurk effect (“+” indicates that such a correspondence exists, while “−” indicates not).

	**Standard -SNN**	**LISNN (Cheng et al., 2020)**	**SOM (Gustafsson et al., 2014)**	**M -SNN**
Simulated McGurk	−	−	+	+
Supervised learning	+	+	−	+

### 4.6. Lower computational cost for M-SNN during training

**Figure 6:**
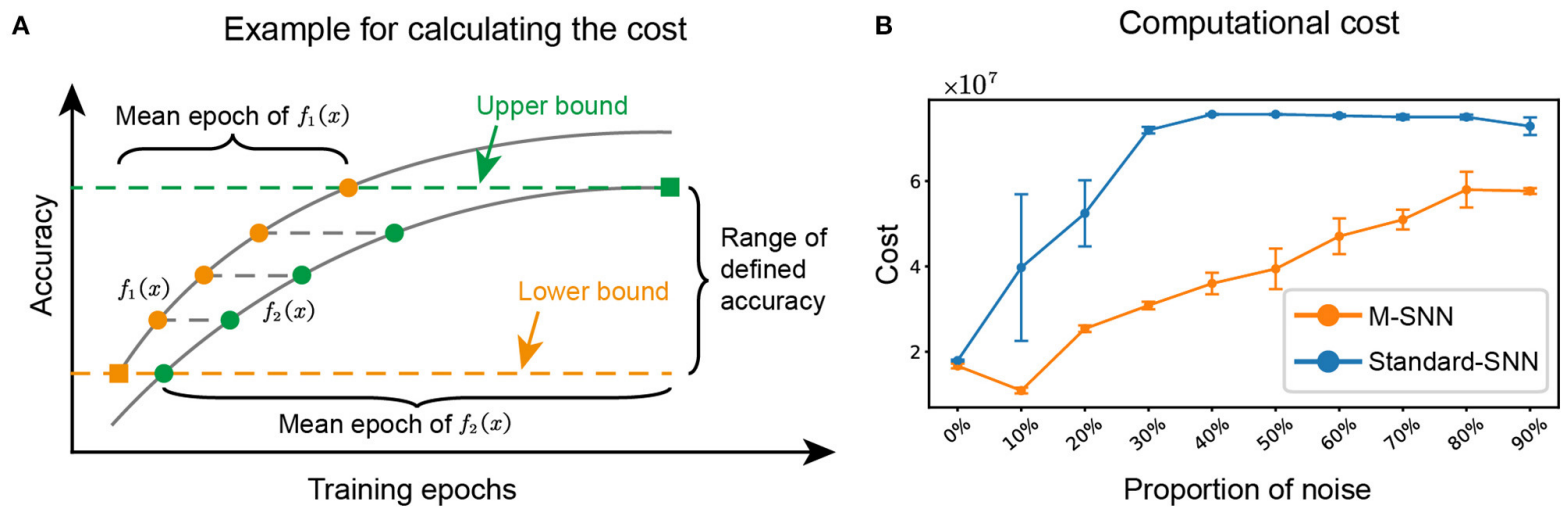
M-SNN in training for lower computational cost. **(A)** Schematic diagram depicting how to calculate the mean epoch during training. **(B)** The computational cost of network training under different proportions of noise.

We referred to the method in paper (Zhang et al., 2021a) to calculate the computational cost of the network during training for algorithm *i*, (*i*=1, 2), where the average training cost of the network was represented by the average epoch multiplied by the number of parameters of the network. A schematic for the mean epoch was shown in Figure 6A, and the equation was shown as follows:


(12) $$\begin{array}{l} Cost_{i}=\frac{1}{N}\overset{N}{\underset{l=1}{\sum }}{\text{Argmin}}_{i}\left(f_{i}\left(x\right)=Acc_{l}\right)\times O{\left(n\right)}_{i}, \end{array}$$

where Argmin_*i*_(·) is the argument when · is the minimum, *f*_*i*_(*x*) is the accuracy function of training epoch *x*, *Acc*_*l*_ is the selected accuracy in [*f*_1_(*x*), *f*_2_(*x*)], *O*(*n*)_*i*_ is the algorithmic complexity of algorithm *i*, and *N* is the number of repetitions. The upper bound is Min[Max[*f*_1_(*x*), *f*_2_(*x*)]] and the lower bound is Max[Min[*f*_1_(*x*), *f*_2_(*x*)]], where Max and Min represents the maximum and the minimum, respectively. In our experiment, *N* = 5 and for the network with *m, n, k* input, hidden, and output neurons, respectively, the *O*(*n*) of M-SNN is (*m* × *n* + *n* × *n* + *n* × *k*) and the *O*(*n*) of Standard-SNN is (*m* × *n* + *n* × *k*).

We calculated the computational cost of training for different proportions of noise. The results of M-SNN and Standard-SNN computational costs were shown in Figure 6B, indicating that the increased noise ratio brought a higher computational cost to the network. In addition, the result showed that the Motifs in M-SNN could save on computational cost when network training (the training cost convergence curves of M-SNN was always below the convergence curves of Standard-SNN). When the noise ratio was 10%, M-SNN achieved the maximum cost-saving ratio of 72.6%. M-SNN achieved the most significant absolute cost savings (save 4.1 × 10^7^) when the noise ratio reached 30%.

## 5. Conclusion

In this paper, we propose a model of Motif-topology improved SNN (M-SNN), exhibiting three main important features. First, M-SNN could improve recognition accuracy in multi-sensory integration tasks. Second, M-SNN could better simulate the cocktail party and McGurk effects than Standard-SNN. Compared with the common Standard-SNN and other SNN methods, M-SNN had a better function of filtering noise from other speakers in different proportions. Furthermore, compared with SNN without Motifs, M-SNN could better handle the McGurk effect with auditory and visual Motif topologies and visual ones. Third, compared with Standard-SNN, M-SNN has a lower computational cost during training in different noise ratios of the background, and the maximum computational cost-saving ratio is 72.6%.

A more profound analysis of the Motifs helps us understand more about the critical functions of the structures in SNNs. This inspiration from Motifs describes the sparse connection in the cell assembly that reveal the importance of the micro-scale structures. Motif topologies are patterns for describing the topologies of a system (e.g., biological cognitive pathways), including the *n*-node meta graphs that uncover the bottom features of the networks. We find that biological Motifs are beneficial for improving the accuracy of networks in visual and auditory data classification. Significantly, the 3-node Motifs are typical and concise, which could assist in analyzing the function of different network modules.

The research on the variability of Motifs will give us more ideas and inspiration toward buildings for a better network. The simulation of different cognitive functions by SNNs with biologically plausible Motifs has much in store to offer in future.

## Code availability statement

The source code can be downloaded from https://github.com/thomasaimondy/Motif-SNN after the acceptance of the paper.

## Data availability statement

The original contributions presented in the study are included in the article/supplementary material, further inquiries can be directed to the corresponding author/s.

## Author contributions

TZ and BX came up with the idea. TZ, SJ, and RZ made the mathematical analyses and experiments. All authors wrote the paper together. All authors contributed to the article and approved the submitted version.
